# Social equity in Human Papillomavirus vaccination: a natural experiment in Calgary Canada

**DOI:** 10.1186/1471-2458-13-640

**Published:** 2013-07-09

**Authors:** Richard Musto, Jodi E Siever, J Cyne Johnston, Judy Seidel, M Sarah Rose, Deborah A McNeil

**Affiliations:** 1 Population and Public Health, Alberta Health Services, Calgary, AB, Canada; 2 Department of Community Health Sciences, University of Calgary, Calgary, AB, Canada; 3 Rho - Sigma Scientific Consultants, Calgary, AB, Canada; 4 Faculty of Nursing, University of Calgary, Calgary, AB, Canada

**Keywords:** Public Health, Human Papillomavirus vaccines, Health equity, Childhood immunization, Deprivation

## Abstract

**Background:**

The Alberta Immunization Program offers a vaccine against the Human Papillomavirus (HPV) free of charge to all girls in Grades 5 and 9. The vaccine is provided in two different service delivery models depending upon the acceptance of the program by the local school board. Vaccinations may be provided “in-school” or in “community” through appointments at Public Health Clinics. The purpose of this study was to determine whether there was a difference in vaccine uptake in Calgary between the two service delivery models, “in-school” and “community”, and to examine if socioeconomic status (SES) was a contributing factor.

**Methods:**

Individual data from the Calgary Zone Public Health vaccination database for all grade 5 and 9 girls in Calgary for school years 2008–2011 were analyzed using descriptive statistics. These data included vaccination records for 35,592 girls. Logistic regression was used to examine the effect of delivery system and SES status on being vaccinated, controlling for school type.

**Results:**

HPV vaccination completion rates were 75% (95% confidence interval = 74.7%, 75.8%) for girls with an “in-school” compared to 36% (95% confidence interval = 35.3%, 37.2%) for girls in schools with a “community” service delivery model. A girl’s neighbourhood SES was related to the likelihood of being HPV vaccinated depending on the service delivery model available to her. For girls attending a Public school with an “in-school” delivery model, the proportion completing vaccination increased as SES decreased (high SES = 79%; medium SES = 79%; low SES = 83%; p-value<0.001). For girls attending Calgary Catholic School District schools with the “community” delivery model there was a decrease in immunization rates from high and mid to low SES (high SES = 41%; medium SES = 42%; low SES = 34%; p-value<0.001). These results show that those with lower SES were differentially disadvantaged by not having access to an “in-school” vaccination delivery model.

**Conclusion:**

Service delivery models make a difference in HPV vaccination completion rates and create inequities for health protection and disease prevention based on socioeconomic status.

## Background

A key goal of Population and Public Health Services is to promote health equity. At minimum this means ensuring universal services, such as immunization programs are designed in such a way as to enhance access by sub-populations understood to be at high risk for the targeted conditions or illnesses. The Alberta Human Papillomavirus (HPV) Immunization Program offers a vaccine that can prevent two types of HPV that cause approximately 70% of all cases of cervical cancer and 90% of genital warts. Since 2008 in Alberta, this vaccine has been offered free of charge to all girls in Grade 5, and since 2009, to all girls in Grade 9. Consistent with the recommendation of the Canadian Immunization Committee, the Program in Alberta was intended to be delivered, like other school-age vaccinations, on school sites [1]. Given that this public health program is delivered in collaboration with the local school districts this also means that the policies should be consistent, and not create barriers for any group.

The goal of promoting health equity appeared to be threatened in 2008 when one of the major publicly funded school districts in Calgary, the Calgary Catholic School District, serving approximately 26% of the population, decided not to permit in-school immunization against HPV. For girls attending schools in this public school district, as well as a small number of private schools that likewise did not permit “in-school” vaccination, an alternate service delivery model for the vaccine was created, namely by appointment at one of the community-based public health clinics (“community” model). It was hypothesized at the time that not only would there be a difference in uptake between the two service delivery models, but that the difference would be particularly pronounced among girls from lower socioeconomic families. The purpose of this study was to determine whether there was a difference in vaccine uptake between the two service delivery models, “in-school” and “community”, and to examine whether socioeconomic status (SES) moderated the effect of service delivery model on vaccine completion.

## Methods

### Setting

Alberta is a western Canadian province with over 3.7 million residents. The province has a single health services delivery system that includes primary, secondary, and tertiary health care services and is divided into five health zones for the delivery and planning of publicly-funded health services. This study was conducted in the Calgary zone, a geographic area that includes the urban city of Calgary and surrounding rural areas. There are three large publicly funded school boards are in this area – The Calgary Board of Education, Rocky View Schools, and Calgary Catholic School District. There are also a small number of Private and Charter Schools. In 2011, the population of the Calgary Zone was 1.4 million [2]. In 2006, the median family income was $82,032, higher than both the provincial and national median family incomes of $76,642 and $66,452 respectively. The Calgary zone also had a higher percentage of the population with university diplomas, degrees, and certificates; 34.9% compared to 26.6% in Alberta and 27.9% in Canada.

### Intervention

In Alberta, recommended vaccinations are provided, solely by Public Health Nurses, without charge, to all children and adolescents with parental consent. Immunizations for school-age children and adolescents are provided within schools during grades 1, 5 and 9, except for the HPV vaccine in some school jurisdictions, where it is available at local community health clinics by appointment. All parents in the province received standardized information on the HPV vaccine, regardless of the vaccine delivery system. Parents in the “Community” vaccine delivery system also received a letter indicating where they could receive their HPV vaccinations.

### Methods and design

A cross-sectional cohort design using administrative data was used for this study.

Calgary Zone Public Health maintains a database to track individual as well as community immunization rates. Nurses and clerks are responsible for entering vaccination data into the database. Initial database entries are derived from live births in the zone and new entries are made when individuals move to the zone and contact Public Health themselves or register their child in school. Clerks also verify that all children enrolled in area schools are included in the database by cross-referencing with school registration data at each immunization time point. A data analyst within Alberta Health Services maintains the database, cleans and verifies data on a continuous basis. This database was used for analysis.

Individual data for all grade 5 (ages 9–11) and grade 9 (ages 13–15) girls for school years 2009–2010 and 2010–2011 was available in the database, as well as 2008–2009 for grade 5 girls. Grade 9 girls did not receive the vaccine in 2008–2009. The database captured 100% of students registered in area schools. Data for girls who were home-schooled and registered with a board of education outside of the City of Calgary were not available. Girls attending Hutterite colony schools (religion-based, rural communities that are isolated from mainstream communities) and those who were home-schooled, but included in the database, were excluded from the study for consistency and the challenge in identifying a vaccination delivery system. Together these girls comprised less than 0.5% of the total sample.

Data included: postal code, school system’s religious affiliation (public [non-denominational], Catholic, and private), grade, vaccination delivery model (“in-school”, “community”), Hepatitis B vaccination status (none, incomplete, complete), and number of HPV vaccine doses received. HPV vaccination status was considered complete if three doses were received within one year; receipt of one or two doses was considered incomplete.

The Pampalon material deprivation index was used as a proxy measure to identify socioeconomic status (SES) for each student based on the geographic location of the student’s residence [3]. Each student’s postal code was linked to the corresponding dissemination area (the smallest standard geographic area for which census data are available and is comprised of 400 to 700 individuals). Statistics Canada 2006 census data on income, education, and employment were used to calculate deprivation factor scores for each dissemination area and the Alberta population was divided into quintiles based on the scores for each area [3]. Each student in our study, based on their residence’s dissemination area, was assigned a deprivation category of 1 (least deprived) through 5 (most deprived) to correspond with highest through lowest SES.

All analyses were performed in Stata S/E Version 12 [4]. Descriptive statistics were conducted with categorical variables expressed as frequencies and percentages, with differences in the distributions examined using chi-square tests. A multivariable logistic regression model was developed using an interactive approach [5] where the outcome variable was complete vaccination (3 doses) versus none/incomplete vaccination (0, 1, or 2 doses). Five variables were considered for entry into the model. The three primary predictor variables were delivery system, school type, and SES. Delivery model and school type were subsequently collapsed into one variable (see below). Hepatitis B vaccination status and grade (as a proxy for age) were also considered because Smith et al. found vaccination history to be the strongest predictor of initiation of HPV vaccination in Ontario, and Reiter et al. found that the daughter’s age was the strongest predictor of parents’ intent to vaccinate [6,7]. For estimation of coefficients and their standard errors, clustering by dissemination area was included to account for correlation between girls living in the same area.

To examine the possibility of interaction and confounding of school type and service delivery model on the relationship between SES and vaccination a new combined variable was created, resulting in the following five school types: public - in-school, private - in-school, private - community, Catholic - in-school, Catholic - community. In addition, due to small cell sizes and similarity of adjacent vaccination rates, some categories of SES were collapsed for logistic regression analysis. The new categories of SES included: High (highest SES category), Medium (second and third categories), and Low (fourth and fifth SES categories).

The possibility of interaction between the two primary variables (delivery model/school type and SES) was considered before inclusion of other variables. The significance of interaction terms was assessed using the Likelihood Ratio Statistic in the model, not accounting for clustering, since an equivalent statistic is not available for clustered data. Interaction terms were considered significant and included in the model if p < 0.05. The remaining predictor variables were eligible for inclusion in the multivariable model if they were significant at p < 0.1 in an individual logistic regression model or if there was evidence of confounding of the primary relationship under observation. Variables were retained in the multivariable model if p < 0.05.

Since this study was cross-sectional in design, it was possible to calculate the estimated proportion vaccinated, along with a 95% confidence interval, for various combinations of the coefficients to aid with interpretation of the final model. The probabilities were generated using the ‘margins’ command in Stata 12, accounting for grade and Hepatitis B vaccination status in these estimates and displayed using a profile plot.

This study was reviewed by the Chair of the University of Calgary Conjoint Health Research Ethics Board and deemed to contain no ethical matters that precluded its conduct.

## Results

**Figure 1:**
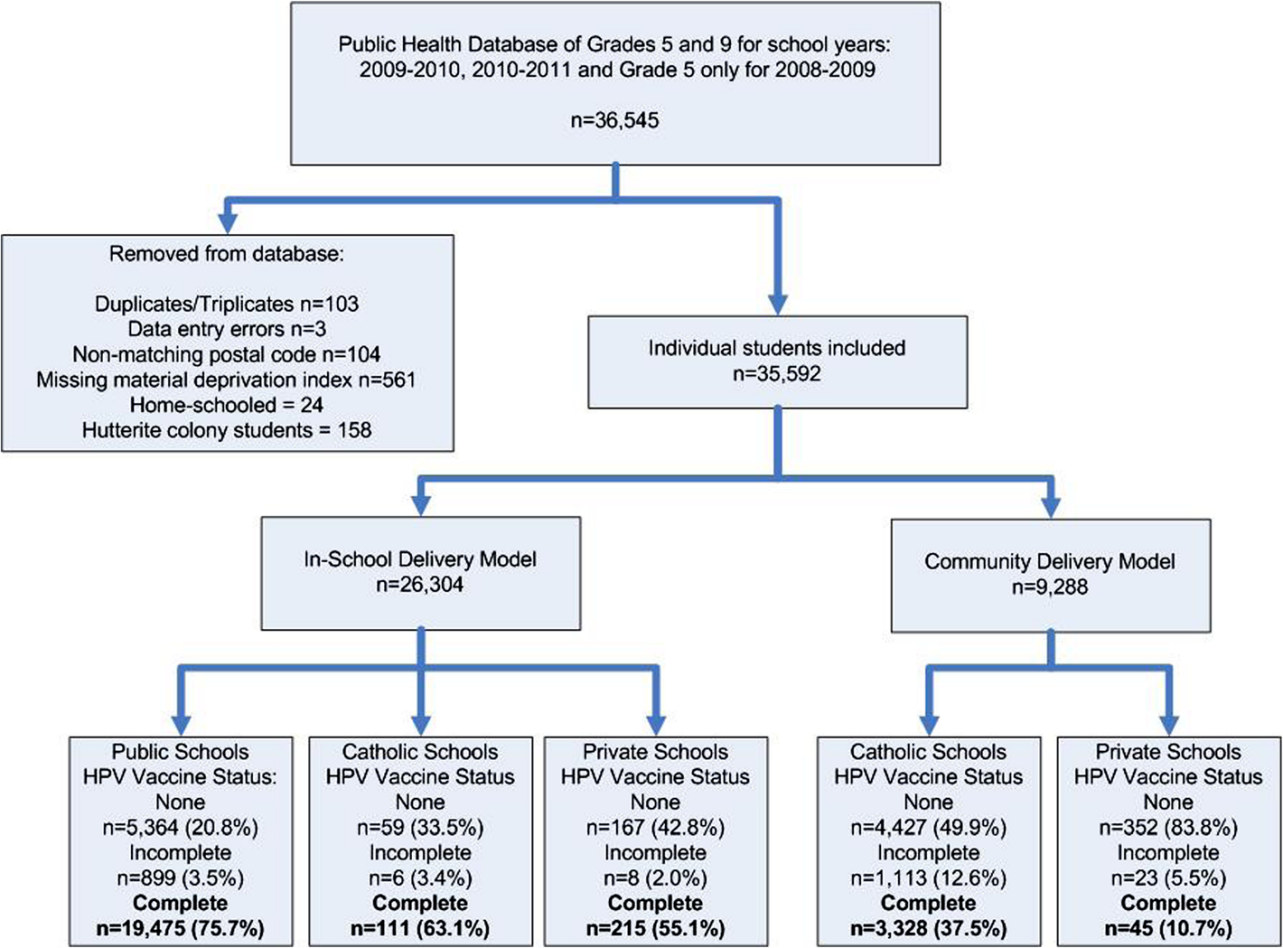
Flow diagram of number of students in the database, reasons for exclusion, and final sample size for analysis (Grade 5 and 9 girls in Calgary Alberta, Canada, 2008– 2011).

The study sample included 97.8% (35,592/36,545) of all girls eligible for the HPV vaccine (Figure 1). Fifty-nine percent (n=20,989) were in Grade 5 (average age of 10.2 years) while 41% (n=14,603) were in Grade 9 (average age of 14.2 years). Ninety-three percent (n=33,098) had previously completed Hepatitis B vaccination. Overall 72.3% of the girls attended Public schools, 25.4% attended Catholic schools and only 2.3% attended private schools (Table 1). There was a significant difference in the distribution of SES by school type within delivery model (p<0.001) for both the “in-school” and the “community” model (Table 1). For example more than half the girls attending the Catholic schools with the “in-school” model lived in neighbourhoods classified as having high SES, compared to less than a third of the girls attending Public schools (Table 1).

**Table 1:** **Distribution of SES by school type for all students in the study** (**Grade 5 and 9 girls in Calgary Alberta**, **Canada**, **2008**–**2011**)

**SES**	**In-school model**			**Community model**		**Overall distribution of SES**
	**Public**	**Catholic**	**Private**	**Catholic**	**Private**	
	**n (%)**	**n (%)**	**n (%)**	**n (%)**	**n (%)**	**N (%)**
1 (High)	7541 (29.3)	91 (51.7)	257 (65.9)	2588 (29.2)	175 (41.7)	10652 (29.9%)
2	5590 (21.7)	37 (21.0)	73 (18.7)	2223 (25.1)	135 (32.1)	8058 (22.6%)
3	4760 (18.5)	22 (12.5)	36 (9.2)	1626 (18.3)	58 (13.8)	6502 (18.3%)
4	3098 (12.0)	13 (7.4)	21 (5.4)	1052 (11.9)	31 (7.4)	4215 (11.9%)
5 (Low)	4749 (18.5)	13 (7.4)	3 (0.8)	1379 (15.5)	21 (5.0)	6165 (17.3%)
Overall distribution of school type N (%)	25738 (72.3%)	176 (0.5%)	390(1.1%)	8868 (24.9%)	420 (1.2%)	35592 (100%)

Overall, HPV vaccination completion rates were 75% (95% CI = 74.7%, 75.8%) for girls with the “in-school” versus 36% (95% CI = 35.3%, 37.2%) for girls in schools using the “community” service delivery model (Figure 1). A girl attending a school offering the “in-school” model was more likely to receive the HPV vaccine than a girl in a school with the “community” model (OR=5.3; 95% CI: 5.1, 5.6) (data not tabled). The vaccination completion rates for each category of SES and delivery model/school type is shown in Table 2 This table illustrates the small cell sizes in some groups as well as the similar completion rates for SES categories 2 and 3, and SES categories 4 and 5 within each delivery model/school type.

**Table 2:** Vaccination completion rates for each SES category and school type (Grade 5 and 9 girls in Calgary Alberta, Canada, 2008–2011)

**SES**	**In-school model**			**Community model**	
	**Public**	**Catholic**	**Private**	**Catholic**	**Private**
	**N=25738**	**N=176**	**N=390**	**N=8868**	**N=420**
1 (High)	5560/7364 (75.5%)	62/90 (68.9%)	155/254 (61.0%)	1000/2255 (44.4%)	21/166 (12.7%)
2	4191/5452 (76.9%)	21/35 (60.0%)	36/69 (52.2%)	882/1982 (44.5%)	17/130 (13.1%)
3	3547/4625 (76.7%)	12/22 (54.6%)	17/35 (48.6%)	654/1436 (45.5%)	2/50 (4.0%)
4	2396/2964 (80.8%)	7/11 (63.6%)	6/21 (28.6%)	362/923 (39.2%)	5/31 (16.1%)
5 (Low)	3830/4539 (84.4%)	9/12 (75.0%)	1/3 (33.3)	430/1159 (37.1)	0/20 (0%)

The logistic regression model revealed a statistically significant interaction between the variables SES and delivery model/school type (LRS=112.89, df=8, p<0.001) in predicting completion of the HPV vaccination. That is, the relationship between a girl’s neighbourhood SES status and the likelihood of receiving HPV vaccination depended on which school type she attended and delivery model available to her. Due to the interaction between the two primary variables of interest in the model, the regression coefficients in the model do not provide a direct interpretation. The full regression model with estimated coefficients is available in the attached Additional file 1: Table S1.

**Figure 2:**
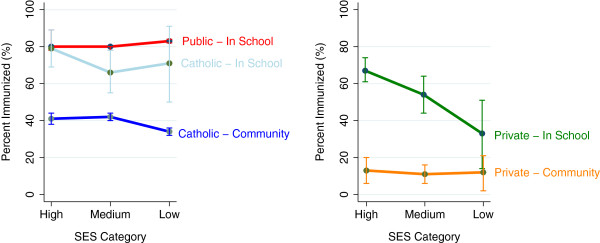
Profile plot with the estimated proportion and 95% confidence interval for completion of HPV vaccine, by school type and SES category (Grade 5 and 9 girls in Calgary Alberta, Canada, 2008–2011).

To aid the reader in the interpretation of this regression model, the estimated proportion of girls completing the HPV vaccine for each of the five school types and three levels of SES (with 95% confidence intervals) are presented in the profile plot in Figure 2. Table 3 provides the numerical values, stratified by prior Hepatitis B vaccination status and SES. Regardless of neighbourhood SES status, girls attending a school with an “in-school” delivery model were most likely to complete vaccination. Conversely, girls attending private schools with the “community” delivery model were least likely to complete vaccination. For girls attending a public school with an “in-school” delivery model, the proportion completing vaccination increased as SES decreased, with 83% (95% CI=82%, 84%) of girls completing the vaccination if they lived in the lowest SES neighbourhoods. Conversely, for girls attending Roman Catholic schools with the “community” delivery model, there was a decrease in immunization rates from medium to low SES, with 34% (95% CI=32%, 36%) of girls completing the vaccination if they lived in the lowest SES neighbourhoods. Seventy-one percent of girls attending Roman Catholic schools with an “in-school” model and living in similarly low SES neighbourhoods completed the vaccination.

**Table 3:** Proportion of complete HPV vaccination by school type, stratified by Hepatitis B vaccination status (Grade 5 and 9 girls in Calgary Alberta, Canada, 2008–2011)

**School type**	**SES category**		
	**High SES**	**Medium SES**	**Low SES**
	Proportion HPV Complete [95% CI]	Proportion HPV Complete [95% CI]	Proportion HPV Complete [95% CI]
Hepatitis B Complete			
Public - In-School	0.79 [0.78,0.80]	0.79 [0.78,0.80]	0.83 [0.82,0.84]
Catholic - In-School	0.78 [0.69,0.89]	0.66 [0.55,0.78]	0.71 [0.50,0.91]
Catholic - Community	0.41 [0.38,0.44]	0.42 [0.40,0.44]	0.34 [0.32,0.36]
Private - In-School	0.67 [0.61,0.74]	0.54 [0.44,0.64]	0.33 [0.14,0.51]
Private - Community	0.13 [0.06, 0.20]	0.11b [0.06, 0.16]	0.12 [0.02, 0.21]
Hepatitis B Incomplete			
Public - In-School	0.18 [0.16, 0.20]	0.18 [0.16,0.20]	0.22 [0.20, 0.25]
Catholic - In-School	0.18 [0.09, 0.27]	0.18 [0.16, 0.20]	0.12 [0.17, 0.23]
Catholic - Community	0.04 [0.03, 0.05]	0.04 [0.03, 0.05]	0.03 [0.02, 0.03]
Private - In-School	0.11 [0.08, 0.14]	0.07 [0.04, 0.09]	0.03 [0.01, 0.05]
Private - Community	0.01 [0.0, 0.01]	0.01 [0.0, 0.01]	0.01 [0.0, 0.01]

Note: Values are estimated from multivariable logistic regression model and control for student grade.

In addition to the significant interaction in the model, grade (p = 0.006) and prior Hepatitis B vaccination (p < 0.001) were significant predictors of complete vaccination. Girls in Grade 9 were more likely to receive the HPV vaccine than girls in Grade 5 (OR=1.02; 95% CI=1.01, 1.03), and girls who received the Hepatitis B vaccination were more likely to complete the HPV vaccination compared to those who did not receive the Hepatitis B vaccine (OR=16.9; 95% CI=14.8, 19.2) (data not tabled).

## Discussion

There was greater vaccine uptake in the “in-school” vaccination delivery model. Controlling for previous Hepatitis B vaccination status and grade, girls who received HPV vaccination in the “community” delivery model were significantly less likely to be vaccinated if they lived in the most materially deprived neighbourhoods. These results support our hypothesis that those with lower SES were differentially disadvantaged by not having access to an “in-school” vaccination delivery model.

It was expected that there would be a difference in the uptake of HPV vaccine between the two service delivery models simply on the basis of the evidence that school-based delivery programs achieve higher coverage rates than community-based delivery by reducing barriers to access [8]. What is not clear, however, is what specific factors contributed to this difference in Calgary. Given that the large dissenting school district is faith-based (Roman Catholic), one possibility might be that the parents actively made the decision to not vaccinate their daughters because of the concerns raised by their religious leaders. This seems unlikely to be the primary factor since we found that girls attending Roman Catholic schools with the in-school delivery were vaccinated at a much higher rate than those attending Roman Catholic schools with the community program, although still slightly less than in the non-denominational schools. Also, in the Roman Catholic school district in Edmonton, another city of similar size in Alberta, permits in-school HPV vaccination, and there was no significant difference in the uptake between the two Edmonton school boards (Alberta Health Services: *Human Papillomavirus Vaccination (HPV) Rates 2009–2010* (unpublished observations)*.* Alberta; 2011). Furthermore, in a provincial survey conducted by Alberta Health and Wellness during the first year of the immunization program, there was no difference found between parents/guardians in Calgary and Edmonton with respect to their intention to consent to the vaccination of their daughters [9]. Finally, several parental surveys have included questions about religious affiliation with conflicting results [10-12]. Ogilvie and colleagues found no difference between reported religious affiliations, while Constantine and Jerman found that both Roman Catholics and non-church attendees were more likely to accept HPV vaccination and ‘others’ and born again Christians were less likely [10,11]. Finally, Marlow, Waller, and Wardle found that parents self-identifying with ‘other’ non-Christian religions were less likely to accept the vaccine than respondents who self-characterized as ‘Christian’ or ‘none’ [12]. There are Catholic students in the Public system and non-Catholic students in the Catholic system in Calgary. We did not have access to the girls’ or their parents’ religious status for either delivery model and therefore were unable to explore this issue further.

The impact of SES has also been somewhat inconsistent in parental surveys. Parents with lower levels of education appear to be more in favour of HPV vaccination than parents of higher education, yet the opposite relationship is seen for parental income; more support for HPV vaccination from parents with higher income compared to parents with low income [13]. In Ontario, Smith et al. found that income was not associated with initiation of HPV vaccination; rather it was associated with completion of vaccination [6]. The authors speculated that absenteeism was an explanatory factor, since if a child missed days when the vaccinations were given in school they were left with scheduling visits either with family physicians or public health clinics. We believe that this is consistent with our findings, and the interpretation that parents who are economically deprived typically face greater challenges getting their daughters to public health clinics than those parents who are economically better off. The Pampalon material deprivation index contains both economic and educational components so we were not able to identify which components contributed to the differences [3].

Vaccine cost is not a barrier to parents in the Alberta program, since the vaccine is provided by the Government of Alberta, and administration of the vaccine is conducted exclusively by Public Health Nurses in Alberta Health Services. However, vaccine cost has been raised as a concern about the program itself. Ironically, the “community” based delivery model in Calgary has proven to be substantially more expensive than the “in-school” model because of the additional staff time required for follow up calls to parents and for vaccine administration (Alberta Health Services: *Costs of Human Papillomavirus Vaccination Services* (unpublished observations) Alberta; 2012). This model also introduces costs to the parents related to transportation, time away from work and inconvenience.

Finally, the information gained through this study is useful for identifying subsets of the population within Calgary who are least likely to access and complete publicly-funded HPV vaccinations. The inequity introduced by the lack of access for girls attending some schools in Calgary is compounded by the fact that another important risk factor for cervical cancer, cigarette smoking, is also more prevalent in these same neighbourhoods and that women of lower SES are known to be less likely to receive cervical cancer screening [14-16]. Following the completion of this study, a combination of factors converged resulting in a change in the delivery of vaccinations within the large dissenting public school district from community to “in-school”. The dissemination of these results to school board members, school and parent councils’ concerns about student access to HPV vaccination, and citizen advocates for HPV vaccination all contributed to this change in policy.

### Limitations

We used an area-based material deprivation index as a proxy measure for individual SES as individual data were not available. While, the material deprivation index has face validity for Calgary, the potential for misclassification of SES and thus misclassification bias cannot be eliminated. Linkage of postal code with the SES data was over 99% thus the risk of selection bias is low. Despite these potential limitations the results of this population based study can inform other jurisdictions planning or examining immunization service delivery models.

## Conclusions

Service delivery models make a difference in HPV vaccination completion rates and create inequities for health protection and disease prevention based on socioeconomic status. Girls living in socio-economically deprived neighbourhoods are more likely to be immunized if they attend schools that have an “in-school” model.

## Abbreviations

HPV: Human Papillomavirus; SES: Socioeconomic status.

## Competing interests

None of the authors have reported any competing interests. Four of the authors are employed by Alberta Health Services, a provincial, publicly-funded health services delivery organization that has set objectives to increase vaccination rates within the province.

## Authors’ contributions

All authors contributed to writing and critically reviewing the manuscript and have given final approval for this version to be published. Specific additional author contributions are as follows: RM contributed in the initial conceptualization of the study and acquisition of the data and its interpretation. JES contributed to the analysis and interpretation of the data. CJ contributed to the design, and interpretation of the data. JS contributed to the design, analysis and interpretation of the data. SR contributed to the analysis and interpretation of the data. DM contributed to the initial conceptualization of the study design and to data analysis, and interpretation.

## Pre-publication history

The pre-publication history for this paper can be accessed here:

http://www.biomedcentral.com/1471-2458/13/640/prepub

## Supplementary Material

Additional file 1 Logistic regression model - The full regression model with estimated coefficients. Click here for file
