# Supplementary material for: Social equity in Human Papillomavirus vaccination: a natural experiment in Calgary Canada
Source: BMC Public Health. 2013 Jul 9;13:640. doi: 10.1186/1471-2458-13-640 (PMC3710270; doi:10.1186/1471-2458-13-640)
Supplement: Additional file 1 — Logistic regression model - The full regression model with estimated coefficients. [file 1471-2458-13-640-S1.docx]

Supplement

Logistic Regression Model

| Complete HPV Vaccination | Coef | Robust S.E. | Z-value | P-value | 95% Confidence Interval | |
| --- | --- | --- | --- | --- | --- | --- |
|  |  |  |  |  |  |  |
| Catholic In-School | 0.024 | 0.310 | 0.080 | 0.939 | -0.584 | 0.631 |
| Catholic Community | -1.677 | 0.066 | -25.280 | 0.000 | -1.807 | -1.547 |
| Private In-School | -0.588 | 0.154 | -3.830 | 0.000 | -0.889 | -0.287 |
| Private Community | -3.188 | 0.311 | -10.260 | 0.000 | -3.796 | -2.579 |
| Medium SES | 0.027 | 0.043 | 0.630 | 0.531 | -0.058 | 0.112 |
| High SES | -0.615 | 0.276 | -2.230 | 0.026 | -1.157 | -0.074 |
| Catholic In-School x Medium SES | -1.622 | 0.053 | -30.560 | 0.000 | -1.726 | -1.518 |
| Catholic In-School x High SES | -1.123 | 0.208 | -5.410 | 0.000 | -1.530 | -0.716 |
| Catholic Community x Medium SES | -3.385 | 0.241 | -14.060 | 0.000 | -3.857 | -2.913 |
| Catholic Community x High SES | 0.287 | 0.054 | 5.310 | 0.000 | 0.181 | 0.392 |
| Private In-School x Medium SES | -0.429 | 0.498 | -0.860 | 0.389 | -1.405 | 0.547 |
| Private In-School x High SES | -1.956 | 0.062 | -31.780 | 0.000 | -2.077 | -1.836 |
| Private Community x Medium SES | -2.024 | 0.424 | -4.770 | 0.000 | -2.854 | -1.193 |
| Private Community x High SES | -3.325 | 0.457 | -7.280 | 0.000 | -4.220 | -2.430 |
| grade | 0.020 | 0.007 | 2.760 | 0.006 | 0.006 | 0.034 |
| hepb | 2.826 | 0.066 | 42.900 | 0.000 | 2.697 | 2.955 |
| Constant | -1.656 | 0.082 | -20.090 | 0.000 | -1.818 | -1.495 |

Log pseudolikelihood = -18924.582

(Std. Err. Adjusted for 1657 clusters in DA)

Number of obs = 35592

Wald chi2(16) = 4321.87

Prob > chi2 = 0.0000

Pseudo R2 = 0.1779
